# Application of machine learning for inter turn fault detection in pumping system

**DOI:** 10.1038/s41598-022-16987-6

**Published:** 2022-07-28

**Authors:** Nabanita Dutta, Palanisamy Kaliannan, Paramasivam Shanmugam

**Affiliations:** 1 Department of Energy and Power Electronics, School of Electrical Engineering, Vellore Institute of Technology, Vellore, 632014 India; 2 Esab India Limited, Chennai, India

**Keywords:** Electrical and electronic engineering, Energy science and technology, Engineering

## Abstract

Pump fault diagnosis is essential for the maintenance and safety of the device as it is an important appliance used in various major sectors. Fault diagnosis at the proper time can reduce maintenance costs and save energy. This article uses a Simulink model based on mathematical equations to analyze the effects of parameter estimation of three-phase induction motor-based centrifugal pumps in inter-turn fault conditions. The inter-turn fault causes a massive in, a massive increase in current, which severely affects the parameters of both motor and pump. These have been analyzed by simulation through the Matlab Simulink model. Later, the results are verified by a hardware in loop (HIL) based simulator. In this paper, machine learning (ML) based artificial neural network (ANN) and ANFIS (ANN and Fuzzy) models have been applied for fault detection. ANN and ANFIS-based models provide a satisfactory level of accuracy. These models provide accurate training and testing results. Based on root mean square error (RMSE), R^2^, prediction accuracy, and mean validation value, these models are compared to find out which is more suitable for this experiment. Various supervised algorithms are compared with ANN, ANFIS, and lastly, found which is the most suitable for this experiment.

## Introduction

The induction motor is a commonly used device indispensable for various industries and has received augmented attention for its robust construction, high performance, reliability, and maintenance cost^1^. Any kind of fault in induction motor causes drastic consequences in the devices connected with the motor and entire system. If pump is connected with a faulty induction motor, the head value will change, the flow rate will change, and colossal vibration will create severe damage^2^. Breakdown of the entire system causes system damage and creates enormous energy loss, and sudden unplanned downtime causes huge maintenance costs. It is reported that 30–40% failure is seen in induction motors for stator inter turn fault^3^. This is actually an electrical fault, and this electrical fault is very sensitive, which causes severe damage. Only 10–20% inter-turn fault causes a massive rising of current in induction motor, which causes insulation losses in the windings^4^. Electrical faults are categorized both as stator and rotor faults^5^. The rotor fault which is seen in the induction motor is a broken rotor bar. Stator faults are mainly three: phase-to-phase fault, inter-turn fault, and phase-to-ground fault. Among those, the inter-turn fault is significant and critical^6^. This inter turn fault hampers induction motor operation and pumping operation. Besides mechanical and hydraulic faults, the electrical fault also hampers the pump performance. A centrifugal pump is a rotating machine used to transfer fluid through pipes. Sudden shutdown of the pumping system causes a huge loss in maintenance^7^. It has been analyzed that 70% of the maintenance cost is seen for the pumping system. So it is required to improve the maintenance technology to reduce cost. Various researches have been done for inter turn fault detection in induction motors. Voltage between lines, neutral and star point of motor were used for fault detection. This was used as model of the motor, and an imbalance was created due to an inter-turn short circuit fault. Before the total breakdown and significant damage to the devices, this imbalance should be identified^8^. Negative sequence impedance was estimated and used as a fault indicator in the research. The negative sequence impendence was seen due to an imbalance in the motor. Oscillation used for Park transformation current was used for fault detection, which was created for imbalance.

To identify this problem, space vector analysis is required^9^. Electrical fault can be detected by motor current signature analysis (MCSA) and vibration analysis. To estimate negative impedance in the motor, robustness with respect to unbalanced voltage supply was added as an approach^10^. Frequency spectrum and fast Fourier transformation (FFT) analysis are also helpful for induction motor fault detection. Wavelet package transformations (WPT) and FFT were used along with some sort of classifier in some work^11,12^.

About faults in the stator for extracting knowledge, higher-order statistics (HOS) was used. For rotating machines, pump health monitoring can analyze supply voltage, current, power, torque, and speed value. Faults in the machine can be analyzed and Identified by harmonic analysis also. In this case, stator current produces instantaneous harmonic current^13^. Parameter estimation technique also performs the pump health monitoring process. Harmonic frequencies can analyze machine health. In hazardous and extreme environmental conditions where access to the machine is difficult such as nuclear power plants, paper mills, chemical plants, and onshore and offshore facilities, this method is applied for health monitoring of the machines^14,15^. MCSA is an electric machinery monitoring technique with wide applications, mainly in heavy industry. It can be used for detecting motor faults. Still, the limitation of this method is it will perform better if it is used in conjunction with other technologies like fast Fourier transformation (FFT), Fuzzy logic, and Park's vector analysis. The latest development in artificial intelligence (AI) is 'transfer learning', which can detect failure patterns of different devices. This technique can be used instead of MCSA to find localized anomalies and has been suggested in place of MCSA for diagnosing bearing faults. Impeller breaking faults in an induction motor^16^. Random forest, support vector machine, extreme machine learning method (ELM), probabilistic neural network have been compared based on various features for rotating machineries fault detection. Based on the accuracy it is find that when data size is limited random forest work better than other algorithms^17^. For the complex system fault diagnosis spatiotemporal pattern network (STPN) with convolutional neural network (CNN) have been used as a hybrid model. This deep learning methods has been used for the analysis of bearing fault data set as a case study. The performance of STPN-CNN has been evaluated based on accuracy rate^18^. CNN and deep adversarial convolutional neural network (DACNN) have been used for mechanical fault diagnosis in a study. The novel DACNN is used to capture domain invariant features. Based on various training samples and 11 case studies gearbox fault diagnosis has been performed^19^. Deep auto encoder has been used for the analysis of various bearing damages in the machines. Scaled exponential linear unit (SELU) has been used to improve the quality of the vibration data for the mechanical fault analysis. Deep transfer technology can be able to make the system more powerful and flexible^20^.

This proposed article presents the stator inter turn fault of three-phase induction motor-based pumping system. The inter-turn fault has been described here as a significant and vital problem in most industrial sectors. Reducing operational and maintenance costs is the main aim of most of the Industries, which increases the importance of condition monitoring of the machine. Requirement of additional equipment and maintenance costs are the burden for industries due to sudden failure of machines. Health monitoring of the winding of the machine is very important to avoid the losses as inter-turn fault causes the damage to the windings. In this research article, overall stator winding fault has been discussed in the next sections, and how the parameters of both motor and pump are affected by stator fault that is also described. As continuous monitoring and prediction of fault detection are possible through ML-based algorithms in a best-suited way, ML algorithm-based ANN and ANFIS models are applied for detecting inter turn stator fault in induction motor-based pumping systems. Both of these models are used in the proposed research, and performances of both algorithms have been compared. Moreover, various supervised algorithms are compared with ANN and ANFIS to find out a better result. Some existing works also have been compared with ANN and ANFIS.

## Proposed model

The proposed research has been done based on some mathematical equations of a pumping system that is coupled with an induction motor. These equations are applied for building the Matlab Simulink model of an induction motor-based pumping system. As fault creation in a real-time system causes significant damage, a mathematical equation-based simulink model has been developed to analyze the healthy and faulty situations. At first, the model was analyzed in healthy conditions without changing any parameter value. Then, to analyze the faulty situation, phase A winding has been sorted such that the current in phase A suddenly increases, and it also helps to increase the phase B and phase C current. Due to changes in current value, torque and speed value also change, and the pump parameters like pressure and flow rate are forced to change as the pump is coupled with the induction motor and runs at the same speed up to three subheading levels are permitted. Subheadings should not be numbered.

## Mathematical model of induction motor-based pumping system

This section describes various equations that express voltage, current, and flux of stator and rotor of induction motor.

1 $$ V_{S} = R_{S} I_{S} + P\lambda_{S} , $$

2 $$ 0 = R_{r} I_{r} + P\lambda_{S} , $$

where

3 $$ V_{S} = [\begin{array}{*{20}l} {V_{as1} } & {V_{as2} } & {V_{bs} } & {V_{cs} } \\ \end{array} ]^{T} , $$

4 $$ I_{S} = [\begin{array}{*{20}l} {I_{as} } & {(I_{as} - I_{f} )} & {I_{bs} } & {I_{cs} } \\ \end{array} ]^{T} , $$

5 $$ I_{r} = [\begin{array}{*{20}l} {I_{ar} } & {I_{br} } & {I_{cr} } \\ \end{array} ]^{T} , $$

6 $$ \lambda_{S} = \left[ {\begin{array}{*{20}l} {\lambda_{as1} } & {\lambda_{as2} } & {\lambda_{bs} } & {\lambda_{cs} } \\ \end{array} } \right]^{T} . $$

*V* represents the voltage, *I* shows current, flux is represented as ${\lambda }_{i}$, here, “*s*” and “*r*” represent stator and rotor respectively, *a*,* b*,* c* denote the three-phase system. *as*_1_,* as*_2_ denote unfaulty and faulty parts of the stator, respectively. Here *P* is the Laplace operator, the derivative operator $\frac{d}{dt}$ is replaced by *P*.

7 $${V}_{as2}= \beta {R}_{s}\left({I}_{as}-{I}_{f}+ \rho {\uplambda }_{as2}\right)= {R}_{f}{I}_{f}, $$

8 $${\uplambda }_{\mathrm{s}}= {\mathrm{L}}_{\mathrm{s}} {\mathrm{I}}_{\mathrm{s}}+ {\mathrm{L}}_{\mathrm{sr}} {\mathrm{I}}_{\mathrm{r}},$$

9 $${\uplambda }_{r}=[{L}_{sr}{]}^{T}{I}_{s}+{L}_{r} {\mathrm{I}}_{r}.$$

These equations show shorted part of stator winding voltage. *β* denotes shorted turn.

The resistance matrix is shown as

10 $${R}_{s}={R}_{s} diag\left[\begin{array}{cc}\left(1-\beta \right) & \beta \end{array} \begin{array}{cc}1& 1\end{array}\right],$$

11 $${R}_{r}= {R}_{r} [I{]}_{3\times 3}.$$

Here, the equations represent mutual inductance and self-inductance of stator winding^12–14^.

12 $${L}_{s}= {L}_{ls} \; diag \; \left[\begin{array}{cc}\left(1-\beta \right) & \beta \end{array} \begin{array}{cc}1& 1\end{array}\right]\left[\begin{array}{cccc}(1-\beta {)}^{2}& \beta *(1-\beta )& -\frac{(1-\beta )}{2}& -\frac{(1-\beta )}{2}\\ \beta *(1-\beta )& (\beta {)}^{2}& -\frac{\beta }{2}& -\frac{\beta }{2}\\ -\frac{(1-\beta )}{2}& -\frac{\beta }{2}& 1& -\frac{1}{2}\\ -\frac{(1-\beta )}{2}& -\frac{\beta }{2}& -\frac{1}{2}& 1\end{array}\right],$$

13 $${L}_{sr}={L}_{ms}\left[\begin{array}{ccc}\left(1-\beta \right)*\mathrm{cos}({\theta }_{r})& \left(1-turn\right)*\mathrm{cos}({\theta }_{r}+2\pi/ 3)& \left(1-\beta \right)*\mathrm{cos}({\theta }_{r}-2\pi/3)\\ \beta *\mathrm{cos}({\theta }_{r})& \beta *\mathrm{cos}({\theta }_{r}+2\pi/3)& \beta *\mathrm{cos}({\theta }_{r}-2\pi/3)\\ \mathrm{cos}({\theta }_{r}-2\pi/3)& \mathrm{cos}({\theta }_{r})& \mathrm{cos}({\theta }_{r}+2\pi/3)\\ \mathrm{cos}({\theta }_{r}+2\pi/3)& \mathrm{cos}({\theta }_{r}-2\pi/3)& \mathrm{cos}({\theta }_{r})\end{array}\right],$$

14 $${L}_{r}=\left[\begin{array}{ccc}{L}_{lr}+{L}_{RM}& -\frac{{L}_{RM}}{2}& -\frac{{L}_{RM}}{2}\\ -\frac{{L}_{RM}}{2}& {L}_{lr}+{L}_{RM}& -\frac{{L}_{RM}}{2}\\ -\frac{{L}_{RM}}{2}& -\frac{{L}_{RM}}{2}& {L}_{lr}+{L}_{RM}\end{array}\right].$$

*β* represents number of turns in phase *a*, ${\theta }_{r}$ represents rotor position, *L*_*s*_ shows self-inductance, *L*_*r*_ shows rotor self-inductance, and *L*_*sr*_ represents stator to rotor mutual inductance. Water is pumped out from a constant level water tank, and the pumping system consists of water tank, an asynchronous three-phase induction motor, and other parts. The tank receives liquid with input flow $\mathrm{represented \; by} {q}_{{v}_{1}}$ Output flow of the control valve is represented by ${q}_{{v}_{2}}$. With the help of fluid mechanics and fundamental physics laws, plant dynamics analysis has been done, and a mathematical model has been developed^21^. This mathematical model includes the mathematical models of centrifugal pump and tank. The counterpart of Newton’s law of force is that angular acceleration is proportional to the torque on the axis. So, the equations show the motion for the motor and pump set.

15 $$J\frac{d\omega }{dt}={M}_{a}-{M}_{p}={M}_{MT}-\left({M}_{p}+\right).$$

*J* shows the moment of inertia. Here moment of inertia is the constant of proportionality in specific case. Active torque of asynchronous motor is shown by ${M}_{MT} \; \mathrm{and \; accleration \; torque \; is \; shown \; by} \; {M}_{a}$, passive or resistive torque of pump is shown by $M_{p}$ and viscous torque is $M_{\zeta }$^22^. Network frequency is shown by *f,* and it is assumed that stator pole pair number is one. Here following equation shows the torque of the asynchronous motor.

16 $${M}_{MT}={k}_{MT}{U}^{2}\left(2\pi f-\omega \right).$$

Viscous torque and passive torque can be represented by

17 $$ M_{\zeta } = k_{\zeta } \omega , $$

18 $$ M_{p} = \frac{{\rho gQ_{v2} H}}{{\eta_{p} \omega }}. $$

Equation 18 shows the basic parameters of the centrifugal pump, and the pump flow rate is shown by *Q*, *H* shows the pump head, and the angular velocity is shown by *ω*. Peripheral cross-section of the impeller channels and meridian component of velocity express the pump flow. Head value is proportional to angular velocity, as the flow rate is proportional to angular velocity^23^.

In the last equation, the pump efficiency coefficient is denoted by which is constant, and in different modes, it changes to some extent, reflecting to the other parameters.

The total operating system $H_{Total}$ can be defined as

19 $${H}_{Total}={H}_{S}+{H}_{D}+\left({P}_{RT}-{P}_{RES}\right).$$

Here the static head is represented by ${H}_{S}$, the dynamic head is shown by ${H}_{D}$, the pressure on the surface of the water in the receiving tank is shown by ${P}_{RT}$, and the pressure on the surface of the water in the reservoir tank is represented by ${P}_{RES}$^24^.

Based on pump height pressure changes and it is considered negligible value. But atmospheric pressure changes with the height. Equation shows the change in pressure and elevation difference between the reservoir and receiving tank. But this is not so significant and considered as negligible.

20 $${P}_{RT}-{P}_{RES}\approx 0.$$

So the equation will be

21 $${H}_{Total}={H}_{S}+{H}_{D}.$$

The difference between the point of discharge and the surface of the reservoir into the receiving tank is the static head which is shown by ${H}_{S}$.The system's static head will vary between maximum and minimum head values because the reservoir's water level also varies.

22 $${H}_{{S}_{min}}=discharge \; level-reservoir \; TWL,$$

23 $${H}_{{S}_{max}}=discharge \; level-reservoir \; BWL.$$

Here top water level is TWL, and the bottom water level is BWL.

Within the system, as a result of dynamic friction head is generated. Basic Darcy Weisbach equation helps to calculate the dynamic head

24 $${H}_{D}=\frac{K{v}^{2}}{2g}.$$

Here the loss coefficient is shown by *K*, velocity in the pipe is shown by and acceleration is $g$.

Now velocity is shown as

25 $$v=\frac{Q}{A }.$$

Here flow rate is shown by *Q* through the pipe, and the cross-sectional area is shown by *A.*

Area *A* is shown as

26 $$A=\frac{\pi {D}^{2}}{4}.$$

The loss coefficient *K* is a form of two elements:

27 $$K={K}_{fittings}+{K}_{pipe}.$$

${K}_{fittings}$ is shown as pumping the water from the reservoir to receiver tank fittings used for the pipeworks of the system.

${K}_{pipe}$ is associated with the length of the pipe, friction, and the diameter of the pipe.

28 $$ k_{pipe} = \frac{FL}{D}. $$

Here *F* shows the friction factor, *L* shows the pipe length, and *D* is the pipe diameter. By the modified version of the Colebrook White equation, the friction coefficient *f* can be found.

29 $$ F = \frac{0.25}{{\left[ {\log \left\{ {\frac{k}{3.7 \times D} + \left. {\frac{5.74}{{{\text{Re}}^{0.9} }}} \right\}} \right.} \right]^{2} }}. $$

Here roughness factor is *k,* and the Reynolds number is *Re*. The roughness factor *k* is a standard fixed value collected from standard tables and depends on the pipe's material and pipe condition. For any flow in the pipe, the following formula is used for the calculation of the Reynolds number^25^:

30 $$Re=\frac{vD}{\vartheta }.$$

$\vartheta$ is the kinematic viscosity. Operation of the pumping system is based on affinity law. First affinity law is shown in the equation where flow *Q* is proportional to shaft speed *N*.

31 $$\frac{{Q}_{1}}{{Q}_{2}}=\frac{{N}_{1}}{{N}_{2}}.$$

As per, the second affinity law, the head is proportional to the square of the shaft speed.

32 $$\frac{{H}_{1}}{{H}_{2}}=\frac{{\left({N}_{1}\right)}^{2}}{{\left({N}_{2}\right)}^{2}}.$$

The power of the pump can be calculated as

33 $$P=\frac{Q\times H\times g\times \rho }{Pump \; Efficiency}.$$

Here *P* is the power requirement for the pump, *H* is the head, $g$ acceleration gravity, and water's density.

## Computer simulation

**Figure 1:**
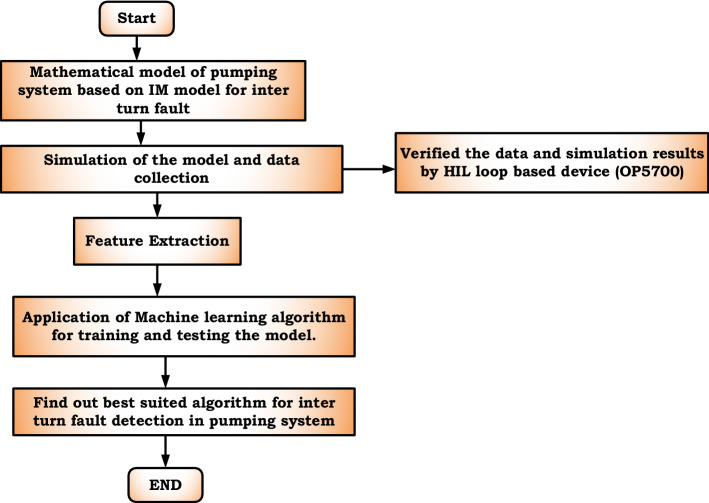
Flow chart of inter turn fault detection in induction motor-based pumping system.

The experiment was done with 3 phase, 50 Hz, 415 V, 0.75 HP squirrel cage induction motor coupled with a VFD-based centrifugal pump with 2800 RPM speed and 23.5-m head value. Under healthy conditions, the three-phase induction motor produces only positive sequence currents and is symmetrical. When symmetry is disturbed during the fault, it generates positive, negative, and zero sequences. The experiment was done by creating an inter-turn fault in the induction motor and analyzing the parameter changes both for the motor and coupled pump. A Simulink model of a three-phase induction motor with turn fault in one phase winding has been built with the help of MATLAB software. The Simulink model has been developed as experimentally it is challenging to create fault due to shorting of high percentage value. After completing the developed model, the model is verified both in healthy and faulty conditions. In different levels of shorting in one phase winding, the model is simulated, and the phase current values are stored in the MATLAB workspace. Negative sequence current, positive sequence current, and zero sequence currents are calculated from these values. The next step is to verify how the inter-turn fault affects various pump parameters coupled with the induction motor. After the simulation process, the results are verified by the OP5700 real-time simulator (hardware in the loop) for validation. In another part of the experiment, ML algorithms have been implemented on simulation data collected through MATLAB to identify and predict faults in induction motor-based pumping systems and analyze which algorithm is suitable for detecting the fault. The Simulink model was built based on the mathematical equations in “Mathematical model of induction motor-based pumping system”. Figure 1 shows the block diagram of inter turn fault detection in an induction motor-based pumping system.

The details of the induction motor are: stator resistance *R*_*s*_ is 0.288 Ω, rotor resistance *R*_*r*_ is 0.158 Ω, stator inductance *L*_*s*_ and rotor inductance *L*_*r*_ is 0.0425 H and 0.0438 H, respectively, mutual inductance *L*_*m*_ is 0.0412 H and inertia *J* is 0.4. Here the number of poles is 2.

Main input parameter shows per unit changes from positive sequence current and negative sequence current for classification of severity of the fault level in phase windings.

34 $$\delta =\frac{(\mathrm{Positive \; Sequence \; Current}-\mathrm{Negetive \; Sequence \; Current})}{\mathrm{Positive \; Sequence \; Current}},$$

$$\mathrm{or} \; \delta =\frac{({I}_{p}-{I}_{N})}{{I}_{p}}.$$

**Figure 2:**
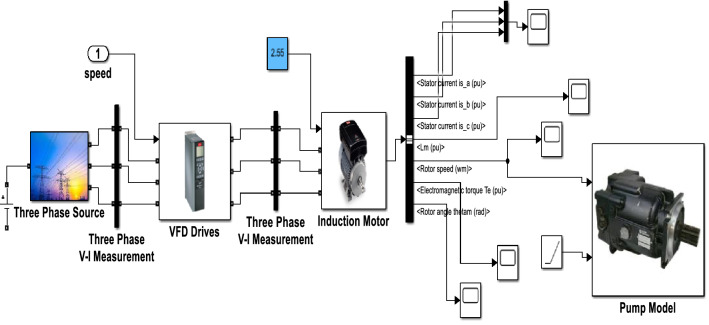
Simulink model of induction motor-based centrifugal pump.

There will be no short circuit turn when the system is in healthy condition. But when the system is in a fault condition, the negative sequence current will increase once the turn fault percentage increases. In the proposed research, up to 40% of the inter-turn fault has been measured. 40%, the value of $\delta \; \mathrm{varies \; from } \; 1 \; \mathrm{ to } \; 0.98, \; \mathrm{ for \; short \; circuit \; level \; of } \; 0 \; \mathrm{ to } \; 40{\%}$ Fig. 2 shows the Simulink model of induction motor-based centrifugal pump system, which has a three-phase source, VFD drives, and an induction motor coupled with a pump. Table 1 shows the magnitude of phase current and sequence component current.

**Table 1:** Magnitude of phase current and sequence component current in different % shortings of Phase A winding.

% Shorting in phase A windings	Phase current value (A)			Sequence components of current values		
	I_a_	I_b_	I_c_	I_positive_	I_negetive_	I_zero_
0	1.54	1.52	1.53	1.601	0.0042	0.0042
1.5	1.62	1.59	1.60	1.628	0.0234	0.02312
2	1.628	1.59	1.60	1.635	0.0238	0.0235
5	1.64	1.59	1.61	1.646	0.0362	0.0369
10	1.641	1.59	1.61	1.679	0.0451	0.0457
15	1.66	1.60	1.615	1.681	0.0628	0.0630
20	1.67	1.60	1.62	1.684	0.0653	0.0654
25	1.68	1.605	1.621	1.691	0.0658	0.0661
30	1.74	1.65	1.63	1.695	0.0671	0.0669
35	1.82	1.68	1.69	1.699	0.0680	0.0670
40	1.85	1.70	1.71	1.7	0.0683	0.0675

Though the simulation model has been analyzed from 0 to 40% short circuit fault, the HIL OP5700 results have been compared between healthy and 40% short circuit fault to check the highest fluctuation during extreme fault conditions. When the inter-turn fault occurs, phase A current increases, and it helps to increase the phase B and phase C current. During fault conditions, the torque response of the motor suffers from oscillations. If the faults occur motor suffers from huge oscillations and when inter turn faults happen motor faces huge oscillations. When the percentage of the turn increases the torque value also will increase and speed value will decrease. The motor is coupled with the pump so that speed is fed to the pump also, and once the pump operates in a fault condition, the flow rate value suddenly increases and pressure decreases. Now, if the pressure goes below the vapour pressure, a cavitation problem will occur, and a sudden increase in flow rate creates a vibrational problem in the overall system.

## Results and discussion

**Figure 3:**
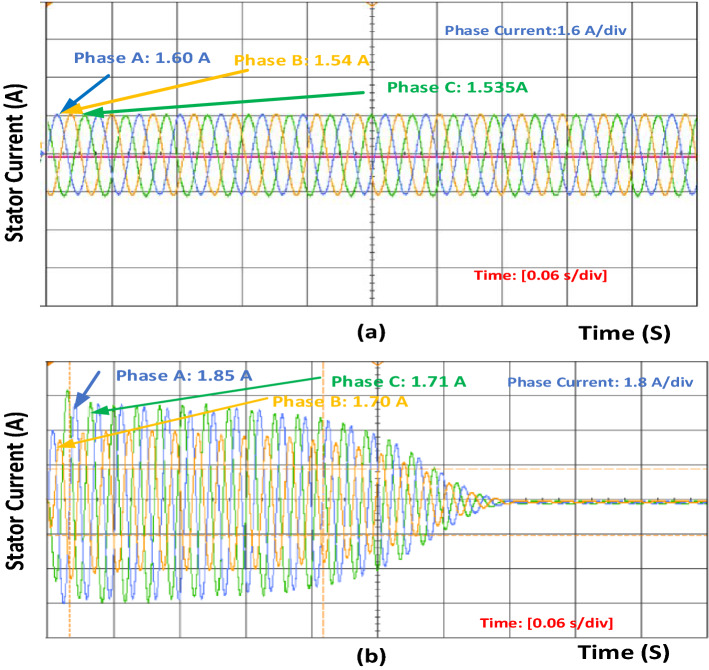
(**a**) Stator current in healthy condition for induction motor. (**b**) Stator current after 40% inter-turn fault in an induction motor.

**Figure 4:**
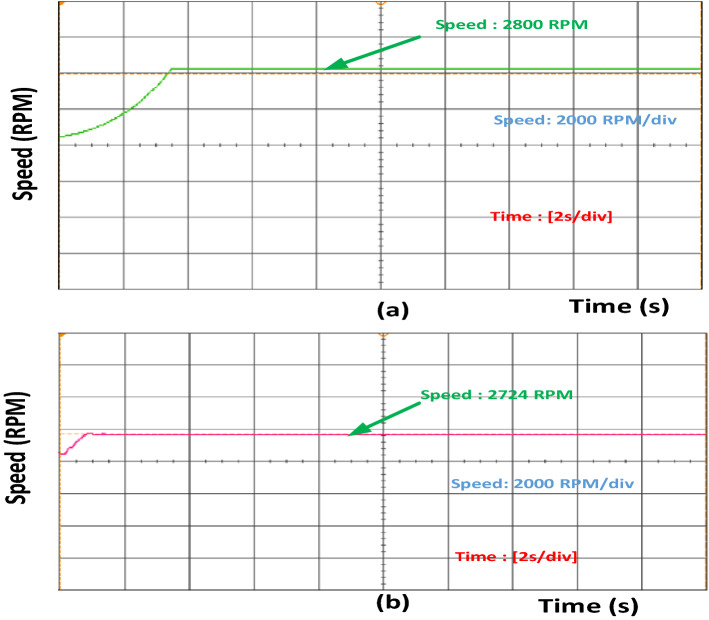
(**a**) Speed for induction motor in healthy condition. (**b**) Speed for induction motor 40% stator inter turn fault occurs.

The figures show the performance curve of current, speed, and torque with respect to time and pump curves for flowrate vs. head value both in a healthy and faulty conditions. All the results were obtained from the OP5700 HIL-based device, which verified the simulation results. Figure 3a,b show the healthy and 40% inter turn fault condition of stator current. Phase A, B, and C current increases as the fault occurs. Figure 4a,b show the healthy and 40% inter-turn fault condition speed value.

**Figure 5:**
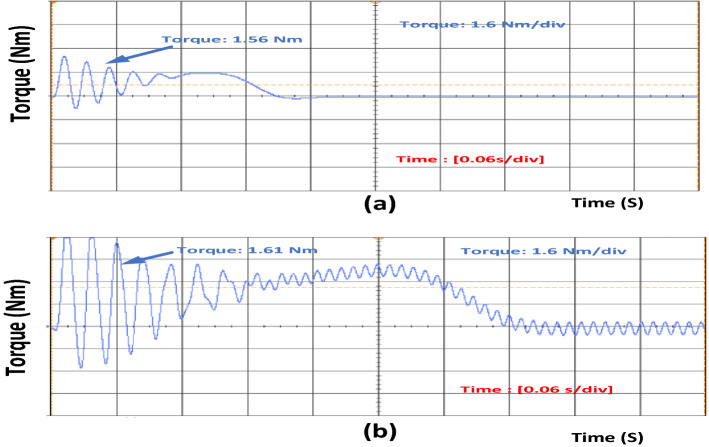
(**a**) Torque of the induction motor in healthy condition. (**b**) Torque of the induction motor in faulty condition.

Similarly, Fig. 5a,b show the healthy and 40% inter-turn fault condition torque value. During a fault, condition motor suffers from oscillations. The size of the oscillations changes when the percentage of turn increases at the same load condition. As the oscillations increase the machine's rated power, the oscillation in the torque also increases.

**Figure 6:**
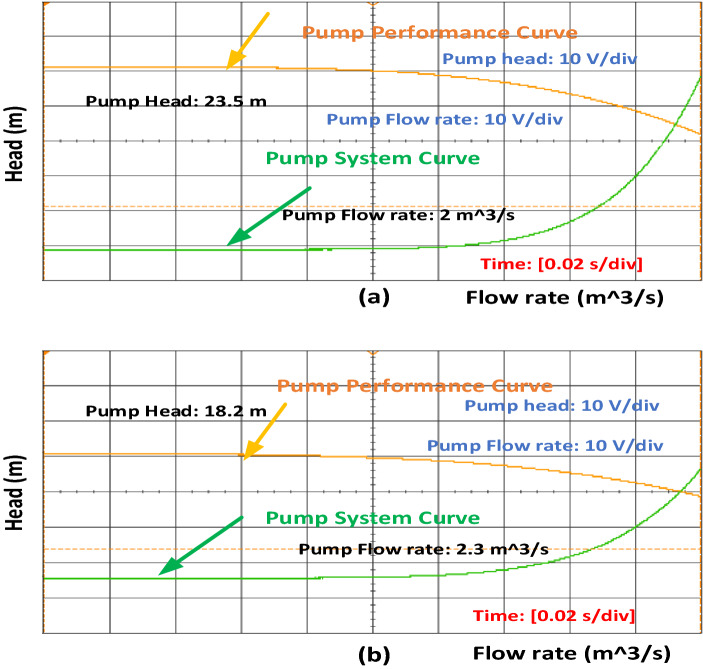
(**a**) Pump performance curve and system curve in healthy condition (**b**) Pump performance curve and system curve in 40% inter-turn fault condition.

**Figure 7:**
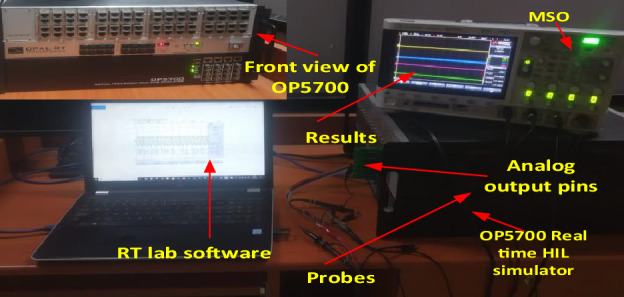
Hardware setup of HIL device.

Figure 3 shows that the current value increases for phase A. Once inter-turn fault occurs and if the number of turns increases, the current value also increases. Increase in phase B and phase C current is also accelerated. Similarly, speed and torque values also increase during fault conditions, as shown in Figs. 4 and 5. Once the motor speed increases, the pump speed also increases. Increase in speed causes an increase in flow rate and a decrease in head value. Figure 6a,b show the pump performance curve and system curve in healthy and 40% inter-turn fault condition. Figure 7 shows the hardware setup of the HIL device.

## Application of ML approach in the induction motor-based pump fault detection

**Figure 8:**
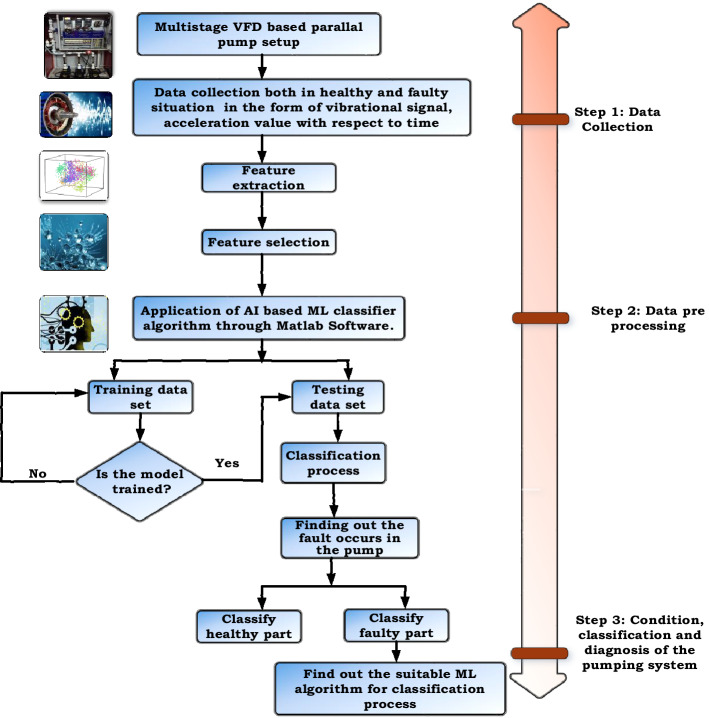
Block diagram of the proposed research for choosing the best-suited algorithm.

Generally, ML algorithms are of two types, supervised and unsupervised, and supervised algorithms have target variables that are formed from the predicted value of input variables. Figure 8 shows the generalized block diagram of the proposed research after the data collection to find out the best-suited algorithm.

The powerful technique ANN is used for the diagnosis of induction motor more accurately. Neural network (NN) is one of the pattern classifiers. Many problems can be solved by using pattern classification of NN k, which involves variable recognition. For induction, motor fault diagnosis it cannot be entirely described or predicted. Mathematical model-based computational algorithm is ANN which behaves like the human brain and thinking process. It has various features like similar parallel processing, self-organizing, self-learning, classification, and non-linear mapping abilities. Combination of Fuzzy and ANN is ANFIS^26,27^, and it is combined to improve speed, fault tolerance, adaptiveness, and to obtain a better modeling system. Based on RMSE, R^2^ values, it can be compared which algorithm is suitable for inter turn fault detection in an induction motor-based pumping system.

The inter turn fault detection in induction motor ANN and ANFIS models are proposed. Artificial immune system for ANN has self-adaptive control and performs better for continuous nonlinear function. The process can be done through online monitoring^28^. ANN is highly interconnected and similar to the human brain and follows a learning process like human beings^29^. Units have interconnections between them and have weights that are multiplied by the values that go through them. Unit has a fixed input known as bias; each unit forms a weighted sum where bias is added. Transfer function analyzes this sum. Prediction of NN depends on training and testing data. The main work of ML algorithm is to make feature extraction. The feature extraction is the important tool which helps to classify the training and testing data for analysis. The most hybrid features are root mean square (RMS), kurtosis value (KV), root amplitude, peak-to-peak value (PPV), standard deviation (SD), skewness value (SV), clearance factor, crest factor (CF), impulse factor (IF), shape factor (SF), and mean value (MV). These statistical features help in the analysis of each signals during healthy and faulty conditions. Feature extraction techniques are used for the statistical analysis for reduction of the large amount of information contained in the current signal which is reflected in the overall signal. The raw current signal is used for the conversion of multiple features for supporting intelligent system to analyse and classify healthy and faulty situation. This overall procedure is called as feature extraction. The statistical features and equations are described in Table 2 which has been used for proposed research.

**Table 2:** Statistical feature based on time.

Features	Equations	Features	Equations	Features	Equations
Root mean square (RMS)	$X_{rms} = \sqrt {\frac{{\sum\nolimits_{n = 1}^{N} {(x(n))^{2} } }}{N}}$	Standard deviation (SD)	$X_{sd} = \sqrt {\frac{{\sum\nolimits_{n = 1}^{N} {(x(n) - X_{m} )^{2} } }}{N - 1}}$	Mean value	$X_{m} = \frac{{\sum\nolimits_{n = 1}^{N} {x(n)} }}{N}$
Peak to peak value (PPV)	$X_{peak} = \max |x(n)|$	Skewness value	$X_{sk} = \frac{{\sum\nolimits_{n = 1}^{N} {(x(n) - X_{m} )^{2} } }}{{(N - 1)X_{sd}^{3} }}$	Shape factor	$X_{shape} = \frac{{X_{rms} }}{{\frac{1}{N}\sum\nolimits_{n = 1}^{N} {|x(n)|} }}$
Crest factor (CF)	$X_{crest} = \frac{{X_{peak} }}{{X_{rms} }}$	Impulse factor (IF)	$X_{impulse} = \frac{{X_{peak} }}{{\frac{1}{N}\sum\nolimits_{n = 1}^{N} {|x(n)|} }}$	Root amplitude	$X_{root} = \left[ {\frac{{\sum\nolimits_{n = 1}^{N} {\sqrt {|x(n)|} } }}{N}} \right]^{2}$
Kurtosis value	$X_{kurtosis} = \frac{{\sum\nolimits_{n = 1}^{N} {(x(n) - X_{m} )^{4} } }}{{(N - 1)X_{sd}^{4} }}$	Clearance factor	$X_{clearence} = \frac{{X_{peak} }}{{X_{root} }}$		

In those equation *x* is the signal and *N* is the no of samples. ANN is powerful techniques by which induction motor faults can be identified. Neural networks are pattern classifiers and are used for pattern classification problems. The most commonly used neural networks are multilayer feedforward network or Levenberg Marquardt method. In the proposed research Levenberg Marquardt method has been used.

**Figure 9:**
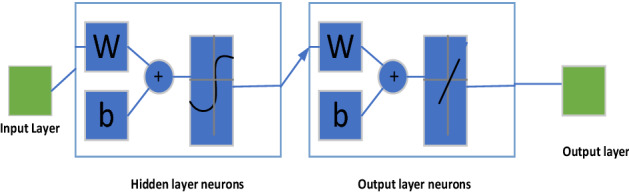
Block diagram of neural network.

The success of training is greatly affected by the proper selection of inputs^30^. Learning process uses testing data and NN constructs input–output mapping. Iteration based on minimization or optimization of some error measured between the output produced and the desired output can adjust the weights and bias. This process is repeated till an acceptable criterion for convergence is obtained. NN consists of the input, hidden, and output layers, as shown in Fig. 9. Output layer consists of six neurons like health condition, 5 turns short circuit, 10 turns short circuit, 20 turns short circuit, 30 turns short circuit, and 40 turns’ short circuit. The algorithm can choose the number of hidden layers by trial and error process.

As the current parameter is the main reason of inter turn fault, so the stator currents are collected in both healthy and faulty conditions such as different shorted turn conditions. Then the currents should be converted in qd frame. The current signals are preprocessed using feature extractions and these features are fed to the classifiers for the diagnosis of induction motor faults. The first case the experiment has been done in healthy conditions and current data have been collected. Then 5, 10, 20, 30 and 40 turn conditions current data have been collected during fault conditions. The three phase currents are converted in qd frame through clerk transformation. The 5000 samples were collected for the each signal duration of 0.2 s. Each signal was divided into 50 segments of 1000 samples. Feature extraction is needed for the processing of raw data signals, then six features were extracted from these segments and as there were two signals so total 12 dimension of dataset has been formed. The total dimension of the dataset is $12 \times 300$. These features were used as input features of neural networks. The dataset then was spitted into training data set, cross validation set and testing data set. The training data set is 70%, 15% for cross validation and other 15% for testing data set shown in Table 3. Training data set was used to train the model and cross validation and testing data set were used to evaluate the performance of the classifier for finding out the accuracy of the model. The means square error was calculated via network to adjust the weight and find the ultimate accuracy rate through training and testing data set.

**Table 3:** Total dataset segment.

Condition	Class label	Total samples	Samples segments	Feature extraction samples	Dataset samples	Training samples	Cross validation samples	Testing samples
Healthy	0	5000	$50 \times 1000$	50	300	210	45	45
5% turn short circuit	1	5000	$50 \times 1000$	50				
10% turn short circuit	2	5000	$50 \times 1000$	50				
20% turn short circuit	3	5000	$50 \times 1000$	50				
30% turn short circuit	4	5000	$50 \times 1000$	50				
40% turn short circuit	5	5000	$50 \times 1000$	50				

**Figure 10:**
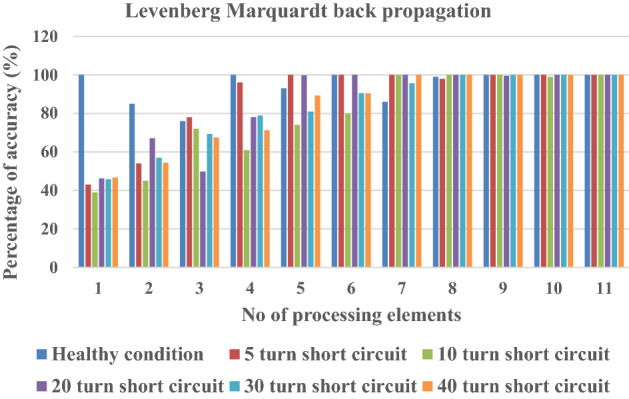
Percentage of accuracy with respect to processing elements in healthy and faulty conditions (training data set).

**Figure 11:**
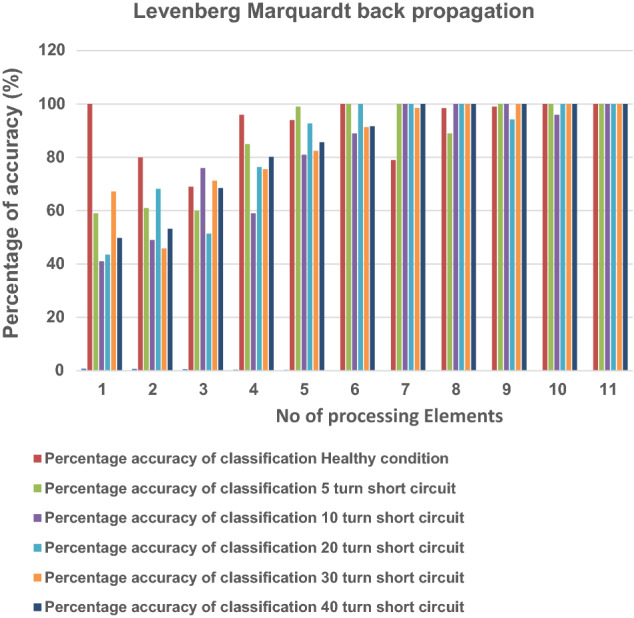
Percentage of accuracy with respect to processing elements in healthy and faulty conditions (cross validation and testing data set).

Levenberg Marquardt back propagation is chosen for training purposes, and training and testing data help obtain the average minimum square error (MSE) for ANN. The average MSE values concerning processing elements present in the hidden layer are shown in Tables 4 and 5. With respect to processing elements, the percentage accuracy of hidden layers are shown for healthy and different turn fault condition in Tables 4, 5, Figs. 10 and 11. In most cases, the Tables 4 and 5 and figures show that accuracy is 100 for healthy and faulty conditions both for training, cross validation and testing dataset.

**Table 4:** Percentage accuracy of classification based on number of processing elements and MSE for ANN (for training dataset).

No of the processing elements	MSE	Percentage accuracy of classification					
		Healthy condition	5 turn short circuit	10 turn short circuit	20 turn short circuit	30 turn short circuit	40 turn short circuit
1	0.691	100	43	39	46.2	45.8	46.7
2	0.540	85	54	45	67.1	56.9	54.3
3	0.439	76	78	72	49.8	69.34	67.5
4	0.231	100	96	61	78.1	78.9	79.2
5	0.195	93	100	74	99.8	80.9	89.3
6	0.0062	100	100	80	100	90.5	90.4
7	1.23e^−2^	86	100	100	100	95.6	100
8	3.67e^−2^	99	98	100	100	100	100
9	4.68e^−6^	100	100	100	99.5	100	100
10	5.78e^−7^	100	100	99	100	100	100
11	6.98e^−8^	100	100	100	100	100	100

**Table 5:** Percentage accuracy of classification based on number of processing elements and MSE for ANN (for testing and cross validation data set).

No of the processing elements	MSE	Percentage accuracy of classification					
		Healthy condition	5 turn short circuit	10 turn short circuit	20 turn short circuit	30 turn short circuit	40 turn short circuit
1	0.760	100	59	41	43.5	67.2	49.8
2	0.652	80	61	49	68.2	45.8	53.2
3	0.592	69	60	76	51.4	71.23	68.5
4	0.381	96	85	59	76.4	75.54	80.21
5	0.245	94	99	81	92.7	82.38	85.64
6	0.00176	100	100	89	100	91.36	91.65
7	1.11e^−2^	79	100	100	100	98.51	100
8	2.61e^−2^	98.5	89	100	100	100	100
9	3.69e^−6^	99	100	100	94.2	100	100
10	4.69e^−7^	100	100	96	100	100	100
11	5.73e^−8^	100	100	100	100	100	100

An intelligent system Neuro-Fuzzy technique ANFIS is used to model and control ill-defined and uncertain systems. Input/output data pairs of the system under consideration build ANFIS. ANFIS is the combination of ANN and Fuzzy, which is used for the learning ability of the fuzzy system. ANFIS consists of five layers^31,32^. Layer 1 is the fuzzification layer which calculates the membership function. Layer 2 represents the rules layer, whose output is the firing strength of each node. Layer 3 highlights the normalization layer, which normalizes the calculated firing strength. Layer 4 shows the consequent layer, whose output layer is the product of normalized firing strength and the fuzzy rules consequent polynomial. Layer 5 shows the overall output and defines the defuzzification layer, whose output is the overall ANFIS output. The problems of continuous changes in mobile learning environments are solved by ANFIS^33^. The proposed ANFIS model can be used for modeling the learner context. Defining input and output values, Fuzzy sets for input values, Fuzzy rules, and creating and training the NN are the steps applying ANFIS to the learner model^34^. Here also stator currents are collected and transformed in qd frame through clerk transformation. The ANFIS model uses discrete wavelet transformation (DWT) or continuous wavelet transformation (CWT). The CWT is a similar concept like FFT but it uses number of wavelets as a function instead of sine and cosine function. The wavelet consists of two parameters like scale and translation and the signal is shown in two dimensional time scale plane instead of one dimensional plane. The Eq. (35) shows CWT function.

35 $$ Wx(a + b;\phi ) = a^{{\frac{ - 1}{2}}} \int {x(t)\phi^{*} \left( {\frac{t - b}{a}} \right)} dt. $$

Here Wx is the wavelet transform linked with two parameters and here a is the scale parameter and b shows as time parameter. ϕ is wavelet function, and *x* (*t*) is the original signal. DWT is used to collect rotor faults and it is mainly used for feature extraction in the proposed research. The ANN and ANFIS models are implemented and compared in the proposed work to find a better performance. R^2^ and root mean square error (RMSE) are used to find out the best-suited model for fault detection of induction motor-based pumping systems. RMSE and R^2^ are used for the analysis of the faults for ANFIS mainly. Based on DWT the performance evaluation can be performed (Table 6).

**Table 6:** The performance evaluation of ANFIS.

RMSE	ANFIS based on DWT	ANFIS
Training RMSE	6.307 × 10^−8^	0.6398
Testing RMSE	6.4321 × 10^−8^	1.1487

Same like ANN for ANFIS also 5000 data samples have been collected and then divided into 100 sections with 500 samples for each sections. These 100 sections are used for feature extraction. 100 samples are used for each condition like healthy or turn fault condition. Totally for six condition 600 samples have been formed. Among these 300 samples have been used for training purpose and 300 samples used for testing purpose (Table 7).

**Table 7:** Data set evaluation.

Condition	Total samples	Samples segments	Feature extraction samples	Dataset samples
Healthy	5000	500	100	600
5%turn short circuit	5000	500	100	
10% turn short circuit	5000	500	100	
20% turn short circuit	5000	500	100	
30% turn short circuit	5000	500	100	
40% turn short circuit	5000	500	100	

Table 8 shows the RMSE value for training and testing data in different conditions.

**Table 8:** ANFIS evaluation in different conditions.

Condition	RMSE of training	RMSE of testing
Healthy	0.154	0.148
5%turn short circuit	0.1765	0.1876
10% turn short circuit	0.2376	0.1854
20% turn short circuit	0.2138	0.1942
30% turn short circuit	0.1287	0.1372
40% turn short circuit	0.2986	0.2561

The performances of the obtained ANN and ANFIS models are also compared after building the model is done. RMSE and R^2^ have comparative statistical values for ANN and ANFIS models, which are given in Table 9. Validation data of the model is 0.05. Prediction accuracy is also measured by R^2^ and RMSE. Prediction accuracy for ANN (R^2^ is 100 and RMSE is 0.054) is better than the ANFIS model (R^2^ is 96.91 and RMSE is 0.121). This the average comparison of total conditions.

**Table 9:** Comparison of RMSE and R^2^ data for ANN and ANFIS.

Parameters	Training data		Testing data	
	ANN	ANFIS	ANN	ANFIS
RMSE	0.054	0.121	0.058	0.062
R^2^	0.998	0.934	0.969	0.897

**Figure 12:**
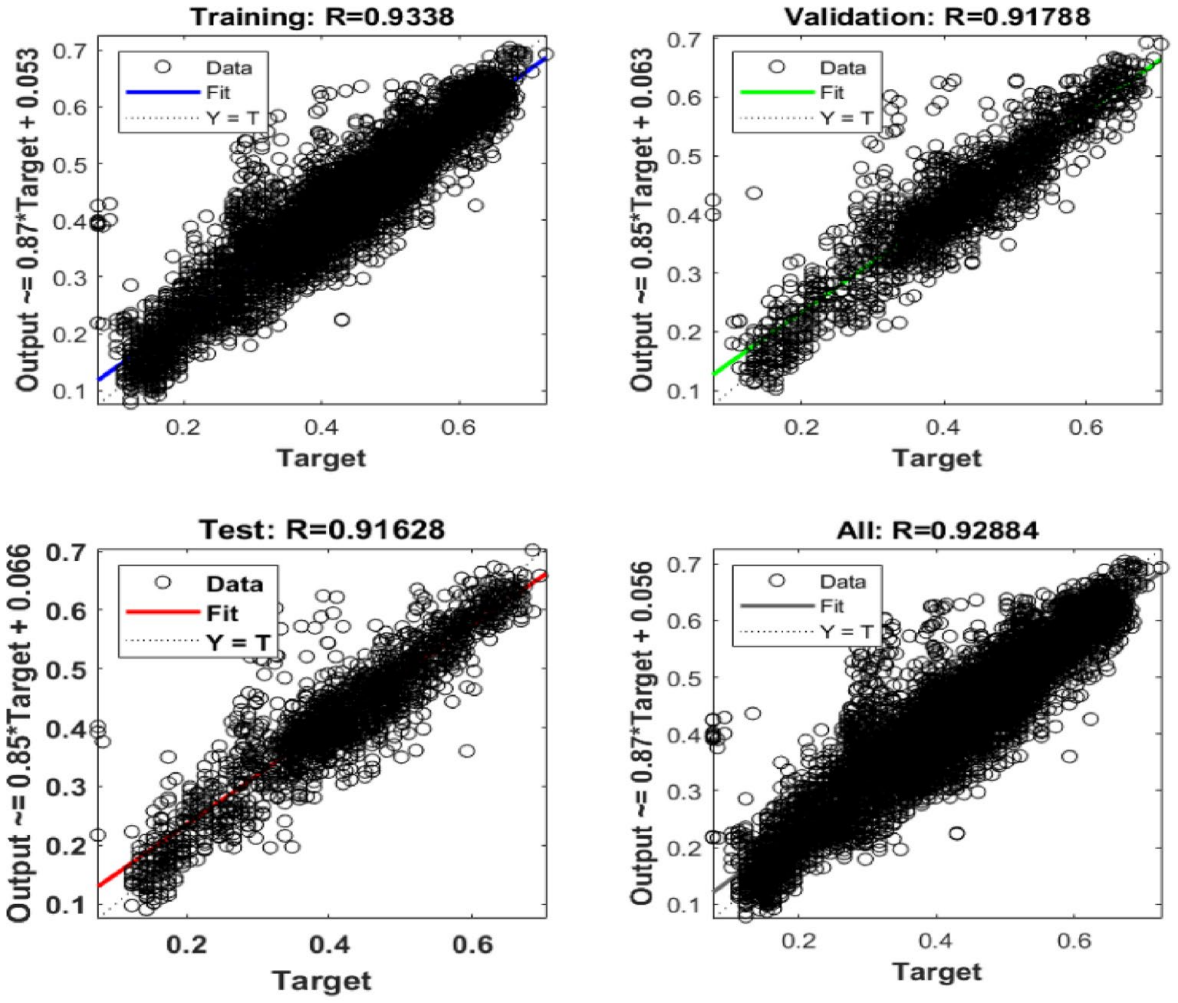
Best fit output of ANN model.

ANN and ANFIS models both performed well and are compatible for fault detection and able to predict the fault. However, based on RMSE and R^2^ of training and testing data, ANN performed better than ANFIS in the proposed experiment. The ANN model has been applied up to 200 epochs, and the best validation has been received in 150 epochs. Figure 12 shows the best fit value of the proposed model of ANN with respect to training, testing, validation and overall values.

## Comparison of ANN and ANFIS with existing studies

Various previous works have been done for inter turn fault detection of induction motor and motor-based pumping systems. Till now, research on the inter-turn fault of induction motor analyzed only changes of induction motor parameters after the fault occurs. But when the motor faces an inter-turn fault problem, the coupled pump is also affected, and the parameters of the pump also change. In the proposed research, including the change of inter-turn fault affected motor parameters, the change in pump parameters also have been analyzed. Current coordinate transform algorithm for inter turn fault analysis in induction motor was developed. Mexbios development studio was built up to analyze the parameter changes in induction motor during a fault condition. Though this is possible to implement in industrial applications, this process cannot predict the fault before the fault occurs and major damages are seen^35^. The proposed model is a simple, easy process and helpful for less amount of data and predict the fault before massive failure. ANN algorithm for inter turn fault detection in induction motor with respect to various turns was used. The experiment created up to 10% inter-turn fault, and phase A current changes were obtained. The experiment analyzed the unit change of positive sequence current from negative sequence current. Experiment was done up to 54 epochs. Here experimental analysis was done for a small level of values^36^. NN model in three different conditions: no load, 50% load, and full load condition for five different motors for inter-turn fault analysis was developed. Up to 15% inter-turn fault was developed, and the accuracy rate of the NN model for various motors varied from 88 to 99%^37^. The novel wavelet analysis was developed in a research.

The model was built to analyze inter-turn fault based on discrete wavelet transformation using Park’s vector transformation. Performance analysis was done for healthy and various turn fault conditions. MSE was obtained for performance accuracy analysis of healthy and faulty situations^38^. Other researches are based on FFT analysis and Park transformation, but these researches are not suitable for predictive control models and not useful for heavy industrial applications^39^. The proposed method of ANN has been used in the current research, which developed the Matlab model to analyze the parameter changes of induction motor and pump during inter-turn fault. As it is the Simulink model, so high range that is up to 40% of inter-turn fault is possible to be created for analysis.

Here ANN and ANFIS models have been implemented in the experimental results, and it is found that the performance of ANN is better than the ANFIS model.

Similarly, the authors also have implemented various supervised ML algorithms like SVM, K-NN, Decision tree, Naïve Bayes, Regression analysis with ANN and ANFIS. Based on accuracy rate, prediction speed, and training time, algorithms are compared to find out most suitable algorithm for this experiment. Generally, ML algorithms have target variables that are to be predicted from independent variables, and these variables generate functions for mapping of input to achieve desired output. After that training process should be done for the better achievement and for more accuracy. The training process is going on until the desired accuracy rate is obtained. No target variable is required for unsupervised learning as it follows the clustering process. SVM is a well-known pattern recognition algorithm mainly used for classification and regression.

## Applications and comparisons of various ML algorithms with ANN and ANFIS

SVM has a hyperplane and margin by separating the dataset and performing the classification task. Optimum hyperplane in the SVM maximizes the width of the hyperplane to avoid overlapping the classes; this is the classification process. Margins are classified between hard margins and soft margins. Since the present diagnosis deals with the non-linear classification problem, a soft margin is used. SVM accuracy depends on three factors: threshold function, cost function, and kernel function. K-NN is a non-parametric versatile learning algorithm also used for classification and regression problems. Instead of learning the discriminative function, the algorithm memorizes the training dataset. By minimizing the training set intense based learning helps to avoid error. The disadvantages of K-NN are ample memory storage, long prediction time, and unnecessary sensitivity to irrelevant features. But when the data size is limited, K-NN works better than any other supervised learning algorithm. A decision tree dendritic classification model is used for both classification and regression problems. Breaking into smaller subsets, the classification process can be done, and based on this, feature selection can be done. The final structure is like tree branches, and each node highlights the feature. Regression analysis provides user equation for graph for the prediction of the data. It always shows the weighted average value for the prediction purpose. Through the statistical analysis it can predict the accurate output. Most elementary statistical courses cover fundamental techniques, like making scatter plots and performing linear regression. The most suitable algorithm can be found based on the overall accuracy rate, prediction speed, and training time.

For the experiment, features are divided into two categories randomly in the ratio of 70:30. In most cases, 70% of data are used for training purposes, and 30% are used for testing data for evaluation. For all the algorithms, the rule is same. The sample data size is 5000 like ANN and ANFIS. For feature extraction purpose 300 data samples have been formed for better analysis. Among these 70% is used for training purpose, 15%for cross validation and rest 15% for testing purpose. The entire diagnosis is carried through the MATLAB pattern recognition and classification learner apps. Based on the evaluation, the accuracy rate of each algorithm is obtained using the formula. With the help of the classification learner app, each algorithm's accuracy rate, prediction speed, and training time have been analyzed and compared.

36 $$Accuracy \; rate \left(\%\right)=\frac{Number \; of \; data \; diagnosed \; properly}{Total \; number \; of \; data \; diagnosed }\times 100.$$

**Figure 13:**
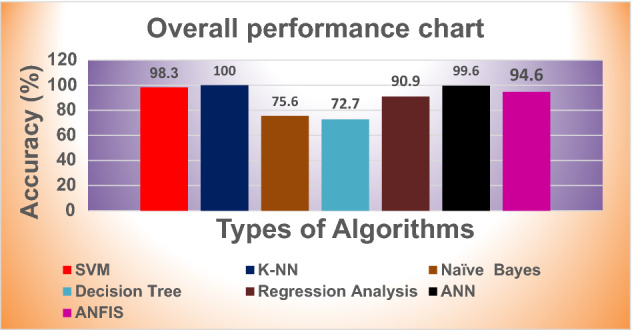
Overall accuracy of various ML algorithms.

From the Table 10 it is seen that performance of the K-NN and ANN are better for this research. But based on accuracy rate, prediction speed and training time K-NN is more suitable than ANN. Figure 13 picturizes the overall accuracy of the ML algorithms.

**Table 10:** Performance analysis of various algorithms.

Algorithms	Accuracy rate (percentage)	Prediction speed (obs/s)	Training time (s)
SVM	98.3	290	0.562
K-NN	100	510	0.063
Naïve Bayes	75.6	410	0.982
Decision tree	72.7	350	1.567
Regression analysis	90.9	467	2.987
ANN	99.6	480	0.086
ANFIS	94.6	320	4.236

## Conclusion

This article explains inter-turn fault analysis of induction motor-based pumping system, and the parameter changes during fault situations in different turn conditions have been shown. The simulation results have been verified through the HIL loop-based (OP5700) device, and the motor's phase current increases when the fault occurs. Once current increases, speed, and torque also increase, affecting the pumping system. Speed helps to increase the flow rate of the pump suddenly, and it causes a huge pressure drop and a decrease in head value. If pressure drops drastically, a cavitation problem occurs, and sudden increase in flow rate causes huge vibration in the pipe, which causes a water hammering problem. In this research, at first, ANN and ANFIS algorithm-based models identification and prediction of the fault have been done. Both the techniques are used, and it is seen that ANN performs better than ANFIS, based on RMSE and R^2^ values. Various other research works are also compared with the proposed work to find out the new development in the proposed work. It is observed that the proposed research is suitable for industrial application and can easily identify the faulty condition for a large amount of data. In the future, the ANN would have been used for other fault detections in motor and pumping systems and for other machineries and can become a comprehensive diagnosis technique. The authors also compared various ML algorithms with ANN and ANFIS, among which, based on accuracy rate, prediction speed and training time it is seen that K-NN and ANN can work better for the proposed research. But based on overall accuracy rate K-NN works better than ANN. In addition, the deployment of the developed technique in a laboratory environment is an extension of the present work. More researches are possible through HIL based OP5700 device to verify the simulation results.

## Supplementary Information

Supplementary Tables.

## Data Availability

All data generated or analysed during this study are included in this published article [and its supplementary information files]. In supplementary file all the data in table form has been added. Further if someone wants to request the data from this study should contact with corresponding author or first author.
