# Supplementary material for: Application of machine learning for inter turn fault detection in pumping system
Source: Sci Rep. 2022 Jul 28;12:12906. doi: 10.1038/s41598-022-16987-6 (PMC9334357; doi:10.1038/s41598-022-16987-6)
Supplement: Supplementary file 1 — Supplementary Tables. [file 41598_2022_16987_MOESM1_ESM.docx]

**Table 1: Magnitude of phase current and sequence component current in different % shortings of Phase A winding**

| **% Shorting in phase A windings** | **Phase Current value (A)** | | | **Sequence components of current values** | | |
| --- | --- | --- | --- | --- | --- | --- |
|  | **I_a_** | **I_b_** | **I_c_** | **I_positive_** | **I_negetive_** | **I_zero_** |
| 0 | 1.54 | 1.52 | 1.53 | 1.601 | 0.0042 | 0.0042 |
| 1.5 | 1.62 | 1.59 | 1.60 | 1.628 | 0.0234 | 0.02312 |
| 2 | 1.628 | 1.59 | 1.60 | 1.635 | 0.0238 | 0.0235 |
| 5 | 1.64 | 1.59 | 1.61 | 1.646 | 0.0362 | 0.0369 |
| 10 | 1.641 | 1.59 | 1.61 | 1.679 | 0.0451 | 0.0457 |
| 15 | 1.66 | 1.60 | 1.615 | 1.681 | 0.0628 | 0.0630 |
| 20 | 1.67 | 1.60 | 1.62 | 1.684 | 0.0653 | 0.0654 |
| 25 | 1.68 | 1.605 | 1.621 | 1.691 | 0.0658 | 0.0661 |
| 30 | 1.74 | 1.65 | 1.63 | 1.695 | 0.0671 | 0.0669 |
| 35 | 1.82 | 1.68 | 1.69 | 1.699 | 0.0680 | 0.0670 |
| 40 | 1.85 | 1.70 | 1.71 | 1.7 | 0.0683 | 0.0675 |

**Table 3: Total dataset segment**

| **Condition** | **Class**  **label** | **Total**  **Samples** | **Samples**  **Segments** | **Feature extraction samples** | **Dataset**  **Samples** | **Training samples** | **Cross**  **Validation samples** | **Testing**  **Samples** |
| --- | --- | --- | --- | --- | --- | --- | --- | --- |
| Healthy | 0 | 5000 | $50\times1000$ | 50 | 300 | 210 | 45 | 45 |
| 5%turn short circuit | 1 | 5000 | $50\times1000$ | 50 |  |  |  |  |
| 10% turn short circuit | 2 | 5000 | $50\times1000$ | 50 |  |  |  |  |
| 20% turn short circuit | 3 | 5000 | $50\times1000$ | 50 |  |  |  |  |
| 30% turn short circuit | 4 | 5000 | $50\times1000$ | 50 |  |  |  |  |
| 40% turn short circuit | 5 | 5000 | $50\times1000$ | 50 |  |  |  |  |

**Table 4: Percentage accuracy of classification based on number of processing elements and MSE for ANN (For training dataset)**

| **No of the Processing Elements** | **MSE** | **Percentage accuracy of classification** | | | | | |
| --- | --- | --- | --- | --- | --- | --- | --- |
|  |  | **Healthy condition** | **5 turn short circuit** | **10 turn short circuit** | **20 turn short circuit** | **30 turn short circuit** | **40 turn short circuit** |
| 1 | 0.691 | 100 | 43 | 39 | 46.2 | 45.8 | 46.7 |
| 2 | 0.540 | 85 | 54 | 45 | 67.1 | 56.9 | 54.3 |
| 3 | 0.439 | 76 | 78 | 72 | 49.8 | 69.34 | 67.5 |
| 4 | 0.231 | 100 | 96 | 61 | 78.1 | 78.9 | 79.2 |
| 5 | 0.195 | 93 | 100 | 74 | 99.8 | 80.9 | 89.3 |
| 6 | 0.0062 | 100 | 100 | 80 | 100 | 90.5 | 90.4 |
| 7 | 1.23e^-2^ | 86 | 100 | 100 | 100 | 95.6 | 100 |
| 8 | 3.67e^-2^ | 99 | 98 | 100 | 100 | 100 | 100 |
| 9 | 4.68e^-6^ | 100 | 100 | 100 | 99.5 | 100 | 100 |
| 10 | 5.78e^-7^ | 100 | 100 | 99 | 100 | 100 | 100 |
| 11 | 6.98e^-8^ | 100 | 100 | 100 | 100 | 100 | 100 |

**Table 5: Percentage accuracy of classification based on number of processing elements and MSE for ANN (for testing and cross validation data set)**

| **No of the Processing Elements** | **MSE** | **Percentage accuracy of classification** | | | | | |
| --- | --- | --- | --- | --- | --- | --- | --- |
|  |  | **Healthy condition** | **5 turn short circuit** | **10 turn short circuit** | **20 turn short circuit** | **30 turn short circuit** | **40 turn short circuit** |
| 1 | 0.760 | 100 | 59 | 41 | 43.5 | 67.2 | 49.8 |
| 2 | 0.652 | 80 | 61 | 49 | 68.2 | 45.8 | 53.2 |
| 3 | 0.592 | 69 | 60 | 76 | 51.4 | 71.23 | 68.5 |
| 4 | 0.381 | 96 | 85 | 59 | 76.4 | 75.54 | 80.21 |
| 5 | 0.245 | 94 | 99 | 81 | 92.7 | 82.38 | 85.64 |
| 6 | 0.00176 | 100 | 100 | 89 | 100 | 91.36 | 91.65 |
| 7 | 1.11e^-2^ | 79 | 100 | 100 | 100 | 98.51 | 100 |
| 8 | 2.61e^-2^ | 98.5 | 89 | 100 | 100 | 100 | 100 |
| 9 | 3.69e^-6^ | 99 | 100 | 100 | 94.2 | 100 | 100 |
| 10 | 4.69e^-7^ | 100 | 100 | 96 | 100 | 100 | 100 |
| 11 | 5.73e^-8^ | 100 | 100 | 100 | 100 | 100 | 100 |

**Table 6: The performance evaluation of ANFIS**

| **RMSE** | **ANFIS based on DWT** | **ANFIS** |
| --- | --- | --- |
| Training RMSE | 6.307x10^-8 | 0.6398 |
| Testing RMSE | 6.4321x10^-8 | 1.1487 |

**Table 7: Data set evaluation**

| **Condition** | **Total**  **Samples** | **Samples**  **Segments** | **Feature extraction samples** | **Dataset**  **Samples** |
| --- | --- | --- | --- | --- |
| Healthy | 5000 | 500 | 100 | 600 |
| 5%turn short circuit | 5000 | 500 | 100 |  |
| 10% turn short circuit | 5000 | 500 | 100 |  |
| 20% turn short circuit | 5000 | 500 | 100 |  |
| 30% turn short circuit | 5000 | 500 | 100 |  |
| 40% turn short circuit | 5000 | 500 | 100 |  |

**Table8: ANFIS evaluation in different conditions**

| **Condition** | **RMSE of Training** | **RMSE of Testing** |
| --- | --- | --- |
| Healthy | 0.154 | 0.148 |
| 5%turn short circuit | 0.1765 | 0.1876 |
| 10% turn short circuit | 0.2376 | 0.1854 |
| 20% turn short circuit | 0.2138 | 0.1942 |
| 30% turn short circuit | 0.1287 | 0.1372 |
| 40% turn short circuit | 0.2986 | 0.2561 |

**Table 9: Comparison of RMSE and R^2^ data for ANN and ANFIS**

| **Parameters** | **Training data** | | **Testing data** | |
| --- | --- | --- | --- | --- |
|  | **ANN** | **ANFIS** | **ANN** | **ANFIS** |
| RMSE | 0.054 | 0.121 | 0.058 | 0.062 |
| R^2^ | 0.998 | 0.934 | 0.969 | 0.897 |

**Table 10: performance analysis of various algorithms**

| **Algorithms** | **Accuracy**  **rate (percentage)** | **Prediction Speed**  **(obs/sec )** | **Training time**  **(Sec)** |
| --- | --- | --- | --- |
| **SVM** | 98.3 | 290 | 0.562 |
| **K-NN** | 100 | 510 | 0.063 |
| **Naïve Bayes** | 75.6 | 410 | 0.982 |
| **Decision Tree** | 72.7 | 350 | 1.567 |
| **Regression Analysis** | 90.9 | 467 | 2.987 |
| **ANN** | 99.6 | 480 | 0.086 |
| **ANFIS** | 94.6 | 320 | 4.236 |
